# Biologically Inspired Model for Inference of 3D Shape from Texture

**DOI:** 10.1371/journal.pone.0160868

**Published:** 2016-09-20

**Authors:** Olman Gomez, Heiko Neumann

**Affiliations:** 1 Institute of Neural Information Processing, University of Ulm, Ulm, Germany; 2 UNITEC, Tegucigalpa, Honduras; Universitat de Valencia, SPAIN

## Abstract

A biologically inspired model architecture for inferring 3D shape from texture is proposed. The model is hierarchically organized into modules roughly corresponding to visual cortical areas in the ventral stream. Initial orientation selective filtering decomposes the input into low-level orientation and spatial frequency representations. Grouping of spatially anisotropic orientation responses builds sketch-like representations of surface shape. Gradients in orientation fields and subsequent integration infers local surface geometry and globally consistent 3D depth. From the distributions in orientation responses summed in frequency, an estimate of the tilt and slant of the local surface can be obtained. The model suggests how 3D shape can be inferred from texture patterns and their image appearance in a hierarchically organized processing cascade along the cortical ventral stream. The proposed model integrates oriented texture gradient information that is encoded in distributed maps of orientation-frequency representations. The texture energy gradient information is defined by changes in the grouped summed normalized orientation-frequency response activity extracted from the textured object image. This activity is integrated by directed fields to generate a 3D shape representation of a complex object with depth ordering proportional to the fields output, with higher activity denoting larger distance in relative depth away from the viewer.

## Introduction

The construction of a neural representation of the 3D shape structure of an object from the monocular 2D information available from the retinal image, is one of the challenging tasks of biological visual systems. The representation of depth structure can be computed from various visual cues such as binocular disparity, kinetic motion and texture gradients. Depth related information can be extracted from a single monocular image from distortions caused on the texture by the object structure and distance of the surface from the camera. The types of distortions are changes in scale, density, orientation contrast and texture compression and anisotropy. The causes for the distortions vary from the type of material of the object, the type of projection, depth differences and the slant and tilt of the surface region. The visual system uses neural sensitivity to gradients of such distortions present in the distribution of neural responses to construct a neural representation of the textured object. Based on findings from experimental investigations [1, 2] we suggest that depth of textured surfaces is inferred from monocular images by a series of processing stages along the ventral stream in visual cortex. Each of these stages is related to individual cortical areas or a strongly clustered group of areas [3]. Based on previous work to develop generic computational mechanisms of visual cortical network processing [4, 5] we propose a model that transforms initial texture gradient patterns into representations of intrinsic structure of curved surfaces (lines of minimal curvature, local self-occlusions) and 3D depth [6, 7]

The ventral stream is one of the two main pathways in the visual system in the primate cortex and it is associated to the visual recognition and identification of objects [8] [9]. It begins at visual cortical area V1 and progresses up to the inferior temporal area (IT), passing cortical areas V2 and V4, among others [10]. The conjunction of areas V1, V2, V4 and IT builds a network of representations and processes that are linked together through feed-forward and feedback connections [11]. Such processes are important for the processing of visual features such as color, shape and texture. Cortical area V1 simple cells have receptive fields with spatial weights which are closely resembled by 2D Gabor filters [12]. The cells’ spatial distribution over the mapped visual field resembles a log polar distribution, where the cells’ response selectivity changes linearly in orientation and logarithmically in spatial frequency [13]. Evidence suggests that one of the tasks performed by the visual system in cortical area V1 is the local frequency decomposition of the image [14].

Another task performed in the visual cortex is the extraction of information from spatial anisotropy for different orientations [15]. Consider for example, a small patch of a surface with texture patterns of isotropic apparent structure on average. Depending on the surface geometry the patch might be slanted away from the viewer in a certain direction. In the spatial domain, the projected texture in the image shows higher anisotropy along the direction perpendicular to the slant direction of the patch. Surface areas with a determinate slant and tilt share a similar spatial anisotropy in a particular orientation. By grouping and enhancing those texture components with similar anisotropy, the slant for that surface region can be estimated and noise, be it in orientation, scale or position, can be reduced. Cells with anisotropic receptive field structure are candidate mechanisms to integrate such oriented structures that are arranged in a specific configuration. It has been suggested that cells in cortical area V2 are involved in such long range integration of oriented input structure [16]. Computational models have formalized such mechanisms by postulating figure-eight shape weightings for oriented long range integration [17, 18]. It has been found experimentally that neurons interact laterally within the same layer with other neurons having the same orientation selectivity through their axon arborizations, which could be modeled as a figure-eight spatial weighting shape, giving a good fit to such bipole cells. They are composed of two sub-fields separated laterally along the major axis, extending from the center in opposite directions. Only when both fields are activated on the input from cells of their corresponding orientation and scale is the cell activated. This results in high response only for those cells for which the input is aligned in orientation.

When the object surface is slanted its texture is compressed. When compared to the texture on a planar surface, this compression causes an increment in the response of the cells to the orientation perpendicular to the direction of slant. The functionality of the bipole cells is then to suppress spurious responses and to detect the alignment of cell responses in a particular orientation, such that when compared with the rest of the orientation responses in the distribution, the anisotropy of the surface region can be inferred. As the texture is distorted, either by the shape of the object surface or its distance with respect to the eye or camera, orientation contrast between local surface regions occurs [5]. By finding the places of high orientation contrast in the image and delineating them, a sketch of the object can be produced. This 2D sketch of the textured object can serve to extract 2D depth special points such as T-junctions or occlusion borders. Such boundaries can occur at different scales and therefore the detection is performed for each spatial frequency band. Model area V4 cells would perform here as edge-line detectors to detect borders between regions grouped by orientation for a particular frequency band. Like area V1, area V4 cells are responsive to different orientations and spatial frequencies. V4 receptive fields consist of overlapping zones that are preferably sensitive to light or dark, like area V1 but larger in size [19]. The cells are involved in the detection of texture boundaries and shape discrimination. In [20], area V4 cells are found to be sensitive to texture density and element size, suggesting the extraction of 3D features from the texture. In our model, it is proposed that area V4 receptive fields can be used for a gradient calculation, which could be fed to the model IT area for the recovery of 3D shape [21].

Cells in area IT are responsive to complex object features. There is evidence for IT cells responsive for texture gradients [2] and complex surfaces of 3D objects [22]. The receptive fields of IT cells are large and cover large part of the visual field. Some of these receptive fields cover only one side of the visual field, with the stronger responses in the foveal region [23–25]. This suggest that IT cells perform an integration of V4 responses to generate a representation of a complex surface. Unlike the fields in V2, which work on the responses of cells in the same orientation, the fields in IT would work in the orientation perpendicular to the orientation of the gradient cells, with the effect of integrating those responses to give a measure of depth.

**Fig 1:**
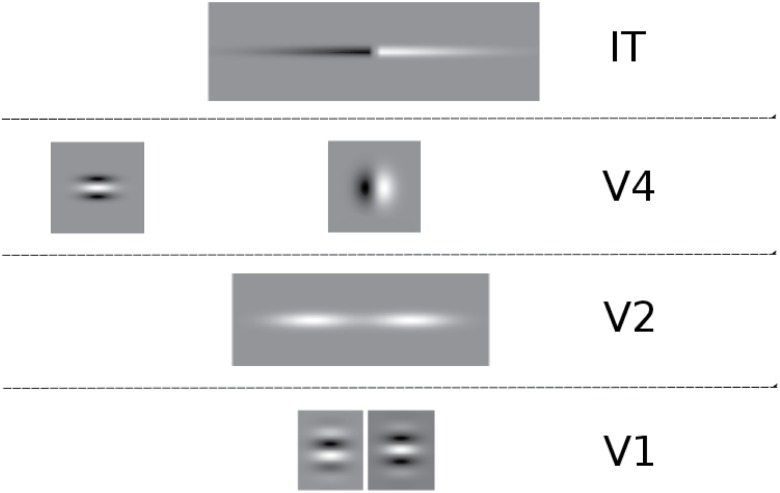
Proposed receptive field shapes as filters for each model cortical area. White is positive and black negative. V1 uses a pair of even/odd Gabor filters. V2 neurons cells axon extensions across orientations are modeled as two weighted fields [18]. The cell is activated strongly only when both subfields have activity. In this way, it is capable of grouping together only those cells responses of similar orientation as well as gap bridging and contour completion. The resulting activity is fed back to the input cell enhancing its response. A similar mechanism is proposed to be performed in area IT, for the integration of texture gradient responses. One sub-field is positive while the other is negative, to interact with the corresponding sign resulting from the V4 Gaussian derivative calculating the change in texture energy. The Gaussian derivative is applied in only one polarity, which makes its resulting activity to have opposite signs when its input activity increases or decreases within its support region with respect to the direction it is being applied. To the far left in V4, the receptive fields used in the calculation of orientation contrast is shown.

Overall, the evidence summarized above suggests that the mammalian neural visual system employs the integration of textural features gradients for the extraction of a three dimensional neural representation of a textured object. In this contribution a neural model is proposed which abstracts the inner workings of cortical areas V1, V2, V4 and IT, to produce two main representations for 3D shape: a 2D sketch of the object and a 3D surface matrix of neural responses corresponding to relative depth values. The shapes of the filters used in the model as receptive fields for each cortical area are shown in Fig 1.

The model developed here adopts the above mentioned mechanisms of frequency decomposition, grouping, texture energy gradient extraction and integration as modules with normalization circuits linked together in a feedforward-feedback chain, generating an abstraction of the processing done in the ventral stream of the visual cortex. The mechanisms make use of the previously described theoretical properties of a three stage cascade of processing in a local model for a cortical column [26]. The two types of representations used by the visual system for 3D shape can be extracted from the model as well as an estimation of the slant and tilt of the surface. Through example output, the plausibility of the model is shown and compared to a ground truth object to test the accuracy.

## Methods

### Model description

One of the aims of the model is to convert the frequency and orientation information from the image to a measure of the relative depth of a surface patch of the object. In order to achieve this the contributions of the neural responses operating in different frequency bands must be clear from noise. This entails the enhancement of the main frequency band responses and suppression of the rest. Higher frequency bands give high responses not only at the edges and contours where they signal high slant but also at edges of texture components, which could lead to noisy results. In order to enable the processing system to automatically adapt and self-calibrate its responses a normalization operation is incorporated which has also been suggested by empirical results [27]. This operation provides stability for the whole process by limiting the value it can reach. Through inhibition in subtractive and divisive form it is possible that the resulting normalization operation causes the enhancement of strong responses while the weaker responses are reduced further. By making this inhibition be composed of pooled responses in the spatial domain as well as feature domain, spurious responses are suppressed not only by nearby stronger cell responses but also from stronger responses in cells responsive to different features. A normalization with respect to mean values within frequency bands prevents the values from increasing without bounds but also maps the values in a manner that takes into account the rest of the responses in spatial and feature space. The model relies on feedback from responses of modules higher in the hierarchy of the architecture back to lower modules for the ordering of depth and border definitions.

### Three stage cascade model

**Fig 2:**
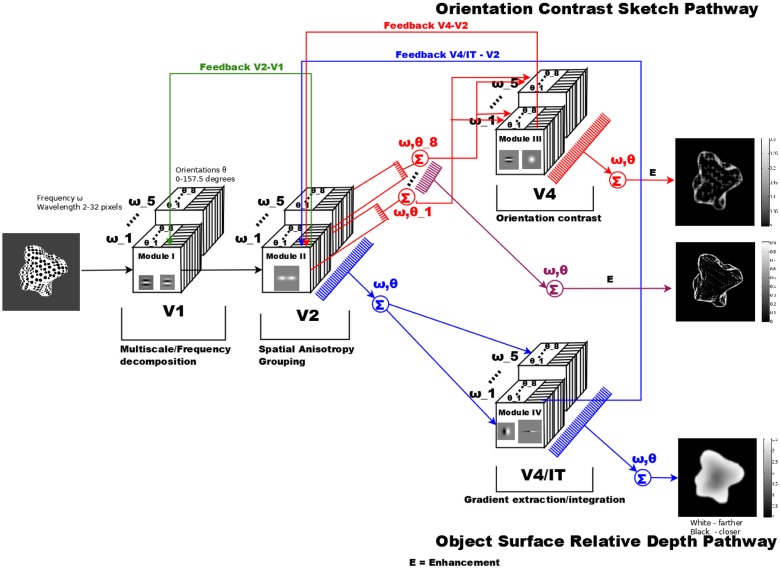
General overview of models schematics. Texture inputs are decomposed into a space-orientation-frequency domain representation. The cascaded processing utilizes computational stages with cascades of filtering, top-down modulation via feedback, and competition and activity normalization. Module I performs a multiple spatial frequency decomposition of the image. These responses are sent to module II, where grouping is performed. The model architecture then splits into two pathways: one for extracting the sketch of the object in module III and the other, module IV, for extracting the relative depth. The responses from each pathways are fed back to module II for border definition and depth ordering. Inside each module box the type of receptive field of the model cells used is depicted.

Our model architecture consists of a multi-stage network of interacting model areas that are coupled bidirectionally [28]. The architecture in Fig 2 is composed of four functional building blocks or modules, each one consisting of three stages corresponding to the compartment structure of cortical areas. This cascade processing model consists of three stages based on the layered processing in the cortex:

- Input filtering, where the feed-forward signals are transformed by linear or non-linear filtering by a mechanism specific to the module.
- Activity modulation of filter outputs by re-entrant signals, where the filtered activity and feedback from higher areas are combined to enhance such activity.
- Center-surround interaction, where both subtractive as well as divisive inhibition from surrounding activity in a pool of cells lead to normalization of activities via space-feature competition.

For an investigation of the dynamic stability properties we refer to [26]. Schematically, the different stages can be formally denoted by the following steady-state equations (with the filter output modulated by feedback and inhibition by activities from a pool of cells (Eq 1) and the inhibitory pool integration (Eq 2)):

$$r_{i,feat}^{I}=\frac{\beta \cdot f\left(F\left(r^{0}\right)\right)\cdot \left(1+net_{i,feat}^{I,FB}\right)-\xi \cdot q_{i,feat}^{I,in}+\eta }{\alpha +\gamma \cdot f\left(F\left(r^{0}\right)\right)\cdot \left(1+net_{i,feat}^{I,FB}\right)+q_{i,feat}^{I,in}}$$ (1)

$$q_{i,feat}^{I,in}=\delta \cdot \left(\underset{feat}{\sum }r_{i,feat}^{I}+\epsilon \cdot \underset{j}{\sum }\underset{feat}{\text{max}}\left(r_{j,feat}^{I}\right)\cdot \Lambda_{ij}^{pool}\right)$$ (2)

where the feedback signal is defined by

$$\begin{array}{c} net_{i,feat}^{I,FB}={\left[\lambda_{FB}-{r_{i,feat}}^{II}\right]}_{+}\cdot \sum_{z\in \{feat,loc\}}r_{z}^{II} \end{array}$$ (3)

**Fig 3:**
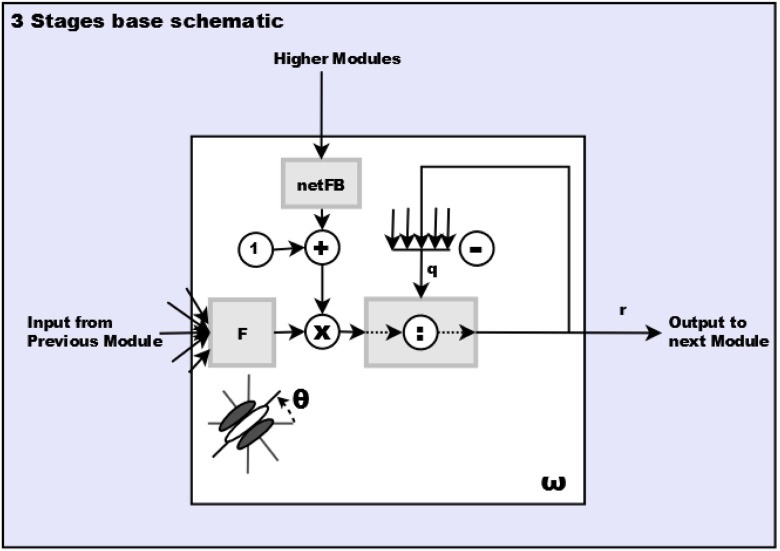
Base schematic for the three stage module in the cascaded architecture. Filtering stage *F* is specific to each module and is applied to input from the previous module. The inhibitory term *q* is composed of pooled activity in space and feature domain. The feedback *netFB* comes from one or more modules higher in hierarchy of the model architecture. The output *r* is likewise sent to one or more modules higher.

The schematic for the generic processing implemented by these equations is shown in Fig 3. The steady-state excitatory equation (Eq 1) with response $r_{i,feat}^{I}$ for a particular image location *i* and set of feature sensitivities (orientation *θ* and spatial frequency *ω*), receives input from earlier module output responses, denoted as *r*^0^ and feedback from one or more modules higher in the hierarchy, denoted as $r_{z}^{II}$. The term $q_{i,feat}^{I,in}$ acts as inhibition, both linearly and divisively, is a weighted sum of the contributions along the feature domain and a spatial neighborhood $\Lambda_{ij}^{pool}$ around the cell location, with parameter *δ* serving as a scaling factor and *ϵ* controlling the importance to the spatial contribution. In order to increase the speed of model response calculations, only the maximum of the responses in the neighborhood are summed. The details for the excitatory, inhibitory and feedback equations are shown in S1B File.

The parameters *β*, *ξ* and *γ* play an important role in the stabilization of the system by keeping the activity within bounds. The resulting activation has an upper bound of $\frac{\beta }{\gamma }$, as the excitatory input and/or the feedback grow very large with respect to the rest of the equation terms. The lower bound when the inhibitory term dominates is −*ξ*. However, negative responses are rectified, using the function *rect*(*x*) = *max*(*x*, 0). The parameter *α* determines the degree of linearity in the response. It can be seen as a form of contrast gain control, with *α* controlling how saturation is reached. The feedback term *net_i_*, *_feat_^I,FB^* corresponds to the total sum in a feature and spatial neighborhood around the point *i*, controlled with the parameter λ_*FB*_ for how much feedback the stage will receive. The parameter values for each module are shown in S1 Table.

Each module implements the three stages of input filtering, modulating feedback and normalization with a particular filter and parametrization according to its feature pair *θ*, *ω*. The filter scale for the modules increases along the architecture. Each module feeds its response to its matching feature pair (*θ*, *ω*) module next in the hierarchy. Interaction across feature space and spatial neighboring cells is done with competition through the inhibitory term $q_{i,feat}^{I,in}$. Between modules the responses could be transformed into a new input, as is done for the different pathways leading to the modules III and IV, where the input is summed over orientations and summed over all domains, respectively (see S1B File). The feedback is also delivered to its matching pair of features (*θ*, *ω*) in modules lower in the hierarchy.

The different stage modules in the model roughly correspond to different cortical areas and the different feature dimensions represented neurally (compare Fig 2): Cortical area V1 computes orientation selective responses using a spatial frequency decomposition of the input; cortical area V2 accomplishes orientation sensitive grouping of initial items into boundaries in different frequency channels to generate representations of surface curvature properties. Different sub-populations of cells in V4/IT are proposed to detect different surface features from distributed responses: One is used to extract discontinuities in the orientation fields (indicative for self-occlusions), another allows anisotropies in the orientation fields of grouping responses to determine slanted surface regions, and one that integrates patches of anisotropic orientation field representations in order to infer local 3D depth.

### Filtering and texture response distributions

The filtering stage *F* uses a particular filterbank for each module and is tuned to different frequency scales and orientations. The filtering is performed on the input *r*^0^ representing the processed output from a lower module in the hierarchical structure. The filters correspond to the receptive fields of the cells of each cortical area that the module maps. Complex Gabor filters [29] as V1 simple cells receptive fields are used in module I. These filters are tuned to different frequencies scaled logarithmically and different orientations scaled linearly. Each filter is used to extract the cell response to a particular frequency and orientation for the image. A pair of separated anisotropic Gaussian weights are used for V2 bipole receptive fields in module II. These filters are also tuned in a log polar manner and are used for grouping their corresponding input pair match (*θ*, *ω*) from module I. Since the subfields responses are multiplicatively combined, only when both subfields in the pair of elongated weights receive high activity from their matching module I responses, their resulting response will be high, denoting alignment of the responses. This results in regions of aligned responses, that is, regions sharing similar anisotropy of the filtered texture pattern. With the modulating feedback and competitive normalization these regions are enhanced.

The response distributions for the different scales tend to look flatter for planar surfaces, as opposed to regions of high anisotropy in projected texture elements. In such image regions maximum activities are generated in module II by filters which are oriented along the axis of high anisotropy. The ratio between maximum and minimum filter responses encodes the slant direction along the axis specified by the filter orientation with the average response amplitude in that region. From the orientation distributions summed along the different scales, a measure of the local surface slant can be computed. This is done through a sum of complex exponentials for each angle of orientation weighted by the corresponding response activity from module II:

$$\begin{array}{c} R=\frac{\sum_{o=1}^{\#\theta }r_{\theta_{o}}^{II,S}\cdot exp(i\cdot \left(2\cdot \theta_{o}\right)}{\sum_{o=1}^{\#\theta }r_{\theta_{o}}^{II,S}} \end{array}$$ (4)

where $r_{\theta_{o}}^{II,S}$ is the sum of each spatial scale band response for orientation *θ*_*o*_. The reason for factor 2 in the exponential is to account for the symmetry in orientation of the filters, where the orientations for the angle of 0 and *π* radians would have the same filter response. By working over the whole 2π domain and considering the filter responses as forces acting upon each other, the 0/*π* orientation response vector affects the rest of the vectors as both initial and end side vector in the complex plane. The angle is calculated by taking the inverse tangent of the imaginary part over the real part of *R*, thus

$$\begin{array}{c} \Theta^{R}=\tan^{-1}\left(\frac{ℑ(R)}{ℜ(R)}\right) \end{array}$$ (5)

and then halved. This gives the orientation which is perpendicular to the slant of the surface, i.e. the axis of the anisotropy. The absolute value of *R*,

$$\begin{array}{c} ∥R∥=\sqrt{\left(ℑ{\left(R\right)}^{2}+ℜ{\left(R\right)}^{2}\right)} \end{array}$$ (6)

gives the magnitude, which is proportional to the slant of the surface with higher values denoting higher inclination of the surface and lower values for more planar surfaces. The anisotropy [15] is another measure proportional to the slant and is calculated through the following formula:

$$\begin{array}{c} A=1-\frac{\min(r_{\theta_{o}}^{II,S})}{\max(r_{\theta_{o}}^{II,S})} \end{array}$$ (7)

The spatial filters for module II respond only when module I responses are aligned in orientation and thus giving a higher response in that orientation and lower response to non-aligned responses. Planar surface areas do not present high alignment for a particular orientation, which make the bipole filter responses similar in value in all orientations. This results in a flat distribution making the second term in Eq (7) close to 1, giving a low value of A while the opposite occurs for slanted surfaces, where one orientation in the distribution dominates.

Module III and IV perform two filtering operations in a cascade organization. For module III, first a filter composed of three alternating anisotropic Gaussians, resembling a second order derivative, is used. This acts as an edge and line detector for the grouped regions per orientation fed forward from module II. Over the result, a difference of Gaussian filter is applied for enhancement. Similarly, in module IV, first a filter composed of two laterally separated anisotropic Gaussians, as an approximation to a first order derivative fields, is applied. This corresponds to receptive fields in cortical area V4 and acts as a gradient calculation on the total sum of the responses fed forward from module II. The filters are also sensitive to different scales and orientation. They detect changes in the grouped frequency decomposition of the texture, where higher values are indicative of larger depth. This is, however, only valid locally, as there is not a global depth ordering and boundaries within the object would have larger values due to larger anisotropy, than adjacent regions. The global ordering is calculated through the directed integration of these gradient responses by the IT filters. For the IT receptive fields, two separated anisotropic Gaussians truncated by 2D sigmoidal functions are used for performing that directed integration of the output responses from the previous filtering stage. One of the fields has negative weights to match the negative sign responses from the input responses due to the Gaussian derivative filters being only applied in one contrast polarity. The Gaussian derivatives can be seen as composed by two subfields, one positive and one negative. The order in which these subfields are applied affects the sign of their response when applied to increasing or decreasing activity within the support of the filter. Since the depth result should always be positive, the sign of the integration result should as well be positive, thus the need for matching sign bipole subfields. These filters have also different scales and orientations. Larger scale filters give a coarse tendency of the depth ordering and shape of the textured object, while smaller scales draw in the depth details. The two fields can be seen as two possible opposite paths of integration for input responses from model area V4 filters. The maximum response of the two integrated activities is chosen. The details of the construction of the filters and related processing can be found in S1A File, S1 Fig.

The approach suggests that the generation of a 2D sketch representation of surface invariants seeks to enhance lines of surface borders, while integrating regions of anisotropy response energy in the orientation domain (over spatial frequencies) allows the inference of qualitative depth from texture gradients. The directed integration of relative change in orientation energy gives the estimate for depth.

### Depth formation

**Fig 4:**
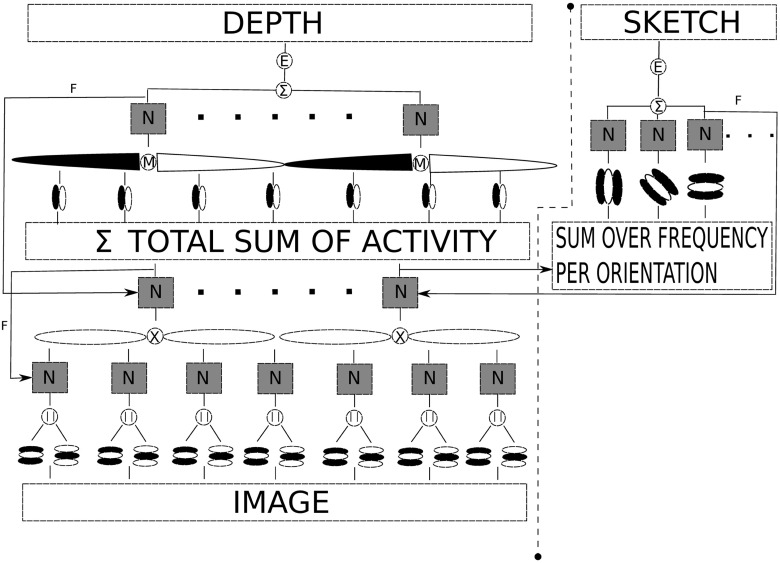
Conceptual diagram of the interactions between the different receptive fields used in the processing of depth and sketch representation, respectively, using the proposed model. N = normalization, || = magnitude, F = feedback, E = enhancement, X = multiplication, M = operation favoring the maximum between the fields lobes responses, Σ = sum.

In this part, the process of depth formation is further explained with images of results for each of the modules in the model. In Fig 4 a diagram showing the simplified interactions of the receptive fields as filters from the model, resulting in the depth and sketch percepts of the object. Starting from the raw image, the model first performs a frequency decomposition with Gabor filters, followed by enhancing that activity which aligns in orientation with bipole gaussian filters, then the total sum of all this activity across frequency and orientation is subjected to a differentiation operation with gaussian derivatives which are the integrated with a pair of opposite signed bipole gaussian filters to give the depth from the sum and enhancement of their resulting combined output.

**Fig 5:**
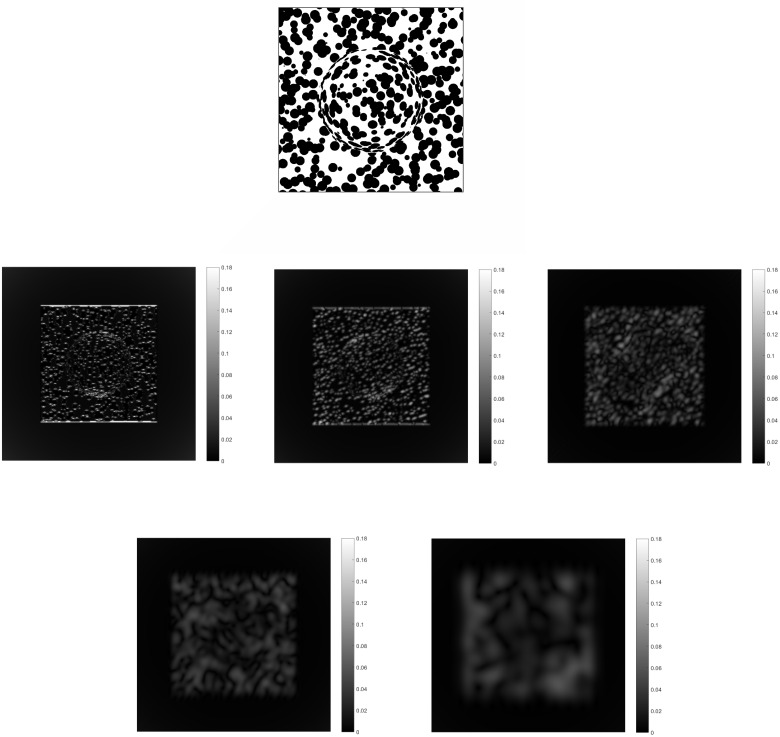
Results from module I for the image of a plane with a semisphere on its center covered with a noisy polka dots texture (top). For brevity and illustration purposes, the module output responses are shown for 5 different orientations from 0 to 90 degrees in increasing frequency selectivity (middle, bottom).

The resulting activity for the modules is obtained for an image of a plane with a semisphere in the middle with a noisy polka dots texture in orthographic projection (Fig 5 top). This is done this way since the scale of the dots would not hint at a depth ordering as is the case in perspective projection, thus showing how the perception of the shape comes from a texture energy gradient integration mechanism. Example results for module I are shown (Fig 5 bottom) for increasing frequency scale and orientation. The texture energy is the resulting amplitude from applying the complex Gabor filters to the textured image. The results at this point look noisy, as the filters respond to all parts of the texture.

**Fig 6:**
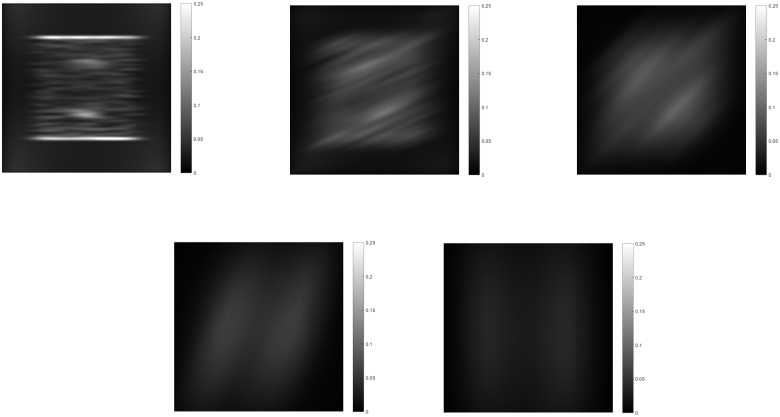
Results from module II for their corresponding module I responses. Those input responses from module I are enhanced only if they are aligned in orientation with the bipole fields.

**Fig 7:**
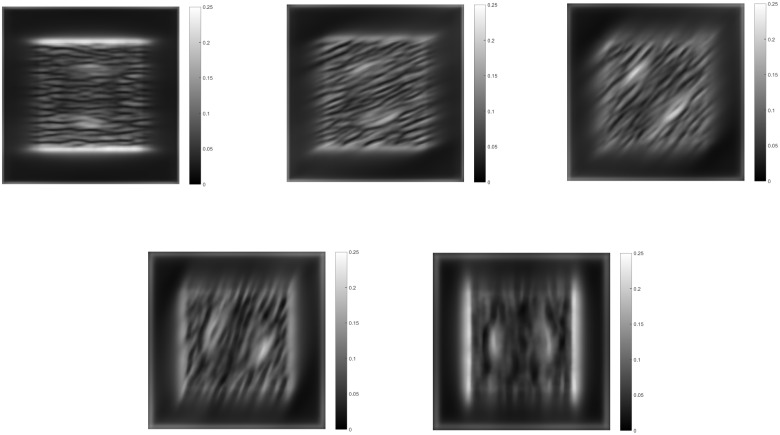
Results from module III. The input activity from module II is used for the extraction of the 2D sketch of the object. Borderlines of regions grouped by orientation similarity are found by the corresponding filters and enhanced through normalization.

Module II takes the corresponding output from module I to find those responses aligned in orientation. The combination of the response of the bipole filters and normalization results in the aligned responses having higher activity. If there is a gap between the responses but they are within the bipole fields reach, the activity at that location would be increased. The results in Fig 6 show this. The more anisotropic a surface patch appears the higher the summed activity would be. By adding all responses from module II for all frequencies and orientations the relative change in depth can be estimated. This is related to the shape of the distribution in frequency space. A higher anisotropy of the texture involves higher frequencies. This implies higher activity from the module II activities, as energy from those higher frequencies is added. For completeness, Fig 7 shows the results from module III for the extraction of the sketch of the image. It detects the changes in regions grouped in orientation.

**Fig 8:**
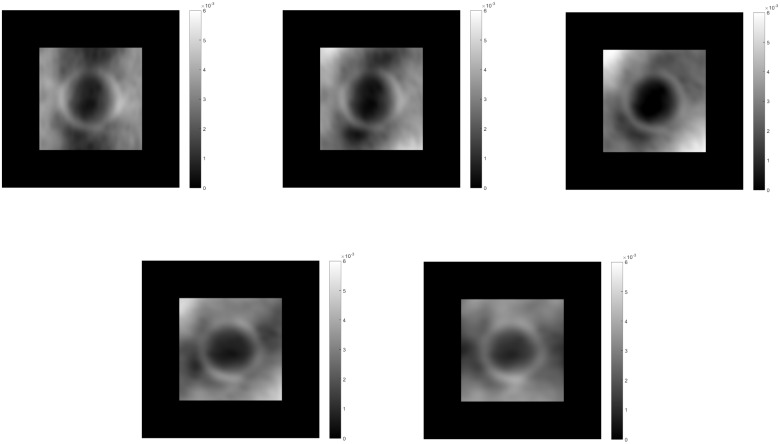
Results from module IV for their corresponding module II responses. The changes in module II summed activity are integrated by the bipole fields resulting in a depth ordering.

Module IV uses first order Gaussian derivatives to extract the changes in the summed activity from module II. It is important to mention that the summed activity is compared with neighboring regions through the Gaussian derivatives. If the summed activity is the same on neighboring regions, the surface will be considered as planar, as the negative and positive fields of the Gaussian derivative cancel out, even when the summed activity comes from anisotropic texture elements. After filtering with Gaussian derivatives, the resulting activity are integrated by two opposing bipole filters. One is positive and the other negative, to interact with the corresponding sign of the resulting Gaussian derivative activity. Then a combination of the fields favoring the maximum is performed, which results in a depth ordering, when the gradient integration spreads. Results for such an operation can be seen in Fig 8 for corresponding orientations. The sum of all these activities gives the final depth ordering.

**Fig 9:**
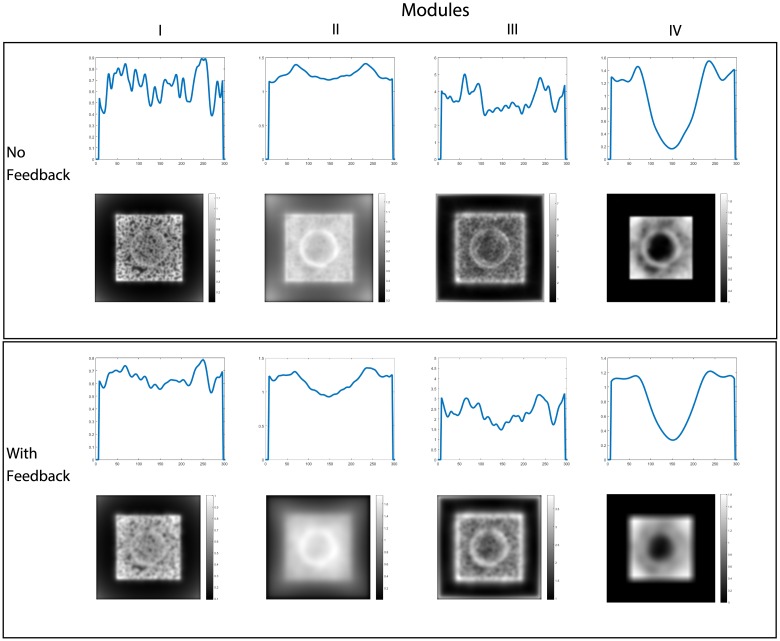
Middle horizontal profiles and results for the total summed activity from each module without feedback(top row) and with feedback enabled(bottom row) for three cycles of iteration. The contribution of feedback can be seen mainly in the profiles of modules II and IV. Without feedback, module II shows ridges at the borders of the semi-sphere, which the integration process in module IV can not completely remove. With feedback, the ridges are reduced resulting in the perception of the semi-sphere over the plane, not as embedded in it.

The feedback signal from module IV is spread down the hierarchy to influence those modules. A comparison of the total summed output of each module with and without feedback is shown in Fig 9. Modules I and III activities show a reduction in noise with feedback. Module II activity shows a reduction in the ridges formed by the high anisotropy of the edges of the semisphere, leading to a more planar shape of its surroundings, giving the correct perceived depth ordering. The contribution of the feedback then results in a more accurate calculation of the gradient and spreading of the depth activity. The resulting activity is then inverted since higher activity is considered as being farther away and is then enhanced with smoothing to establish the shape. Normalization in context of space, orientation and frequency in all modules limits the depth estimation from growing without control.

**Fig 10:**
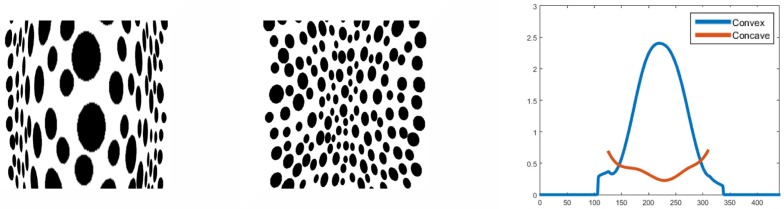
Texture surface patches with bell-shaped convexity (left) and bell-shaped concavity(middle). Both surface patches are seen from the front and from behind in perspective projection. The perceptual appearance of the concave patch is like a narrow ridge in comparison to the convex surface appearance. The model computations replicate this in a qualitative fashion. Middle horizontal profiles for the inverted summed activity from module IV are shown for both input shapes(right). The profile of the first image is seen convex (blue) and for the second (red) as concave. The model slightly underestimates the depth of the bell shape for the second image and the profile looks shallower compared to the first.

This figure with the semisphere is ambiguous, as it could be interpreted as either a convexity or a concavity. The model would tend to prefer convexity, going from areas of low activity to those of higher activity. Fig 10 shows an example of resulting profiles for a bell shaped elongated object covered with a polka dots texture in perspective projection. The size of the bipole filters in module IV should be proportional to the object size to cover the surface so that a correct integration is made. The size of the bipole fields in module II similarly should be large enough to cover gaps in the texture to correctly assess the alignment. Otherwise, large gaps could be interpreted as bumps, given the low frequency activity there.

## Results

**Fig 11:**
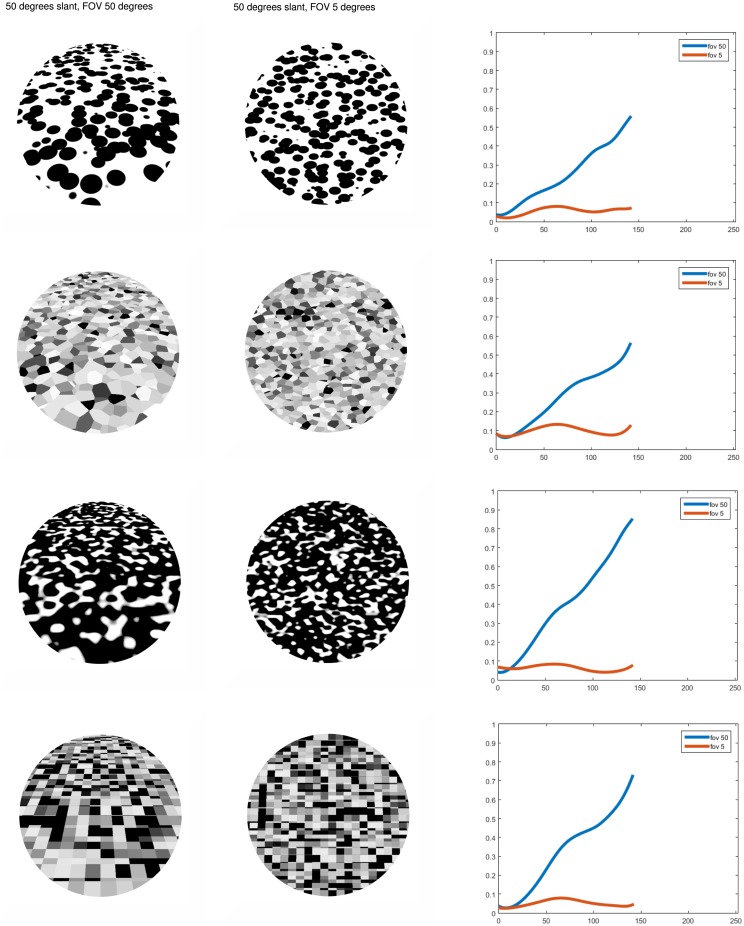
Profiles for the middle vertical line of results for images of a plane slanted 50 degrees with different textures with fov of 50 and 5 degrees.

In order to demonstrate the functionality of the new proposed model architecture we show below several results. Grayscale images of size 300 by 300 pixels where used for different challenging textures covering objects with complex surfaces. The images were produced in the 3D software Blender with the procedural textures available. The model produces two types of final output results: A 2D sketch showing object boundaries and occlusion borders; and a 3D mesh with values indicating the relative depth of the surface location, with low activity response corresponding to the surface being closer to the viewer and farther for higher values. First, in Fig 11, the model is applied to images of slanted planes for different noisy textures. The plane is seen with 50 degrees of slant and the images are taken under perspective projection with a field of view(fov) of 50 and 5 degrees. The results show that even when the profiles show small undulations due to the noise in the texture, the inclination of the plane is captured for the 50 degrees fov. For the 5 degrees fov the profiles looks planar, even when the aspect of the texture is anisotropic. This is due to there being no change in total orientation aligned texture energy with respect to neighboring surface regions and thus giving a low activity gradient calculation and subsequent integration. Such perception phenomenon is reported in [30] for human subjects.

**Fig 12:**
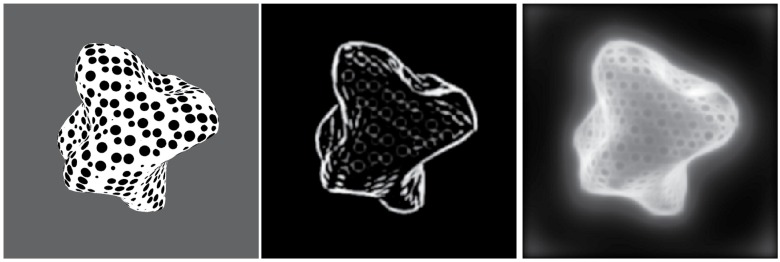
Result of grouping initial filter responses in space-orientation domain (separately for individual frequency channels) for the input image (left). The maximum responses over frequency and orientation (white) create a sketch-like representation of the ridges of a surface corresponding with the orientation of local minimal curvature (middle). Also local junctions occur due to self-occlusions generated by concave surface geometry. Texture gradient information is calculated over the normalized grouped responses of cells in different frequency channels in module II (right). Stronger response anisotropies are mapped to white. The short axis of the anisotropies (strongest compression) coheres with the slant direction.

**Fig 13:**
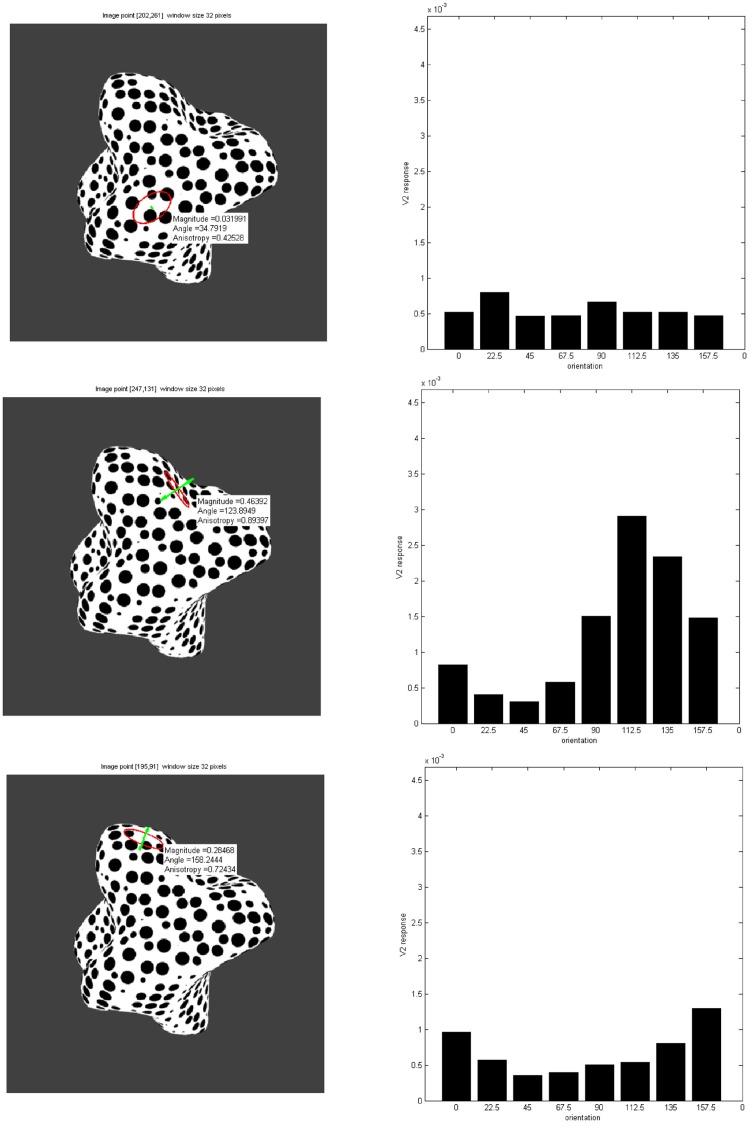
Three surface locations are shown with their respective distribution of summed responses per orientation from module II, shown as histograms(right column, each bin value corresponds to the summed activity over frequency for that orientation). Magnitude, angle and anisotropy values of the texture pattern at that point are calculated from this distribution. An ellipse is drawn to represent the anisotropy on the surface. The major axis corresponds to the maximum value in the distribution and the minor axis to the minimum. A green line is drawn perpendicular to it to show the resulting orientation of slant (there is an ambiguity in the direction of slant since the surface tilt can be along opposite directions locally). Planar surfaces show low anisotropy and magnitude values while slanted surfaces show high anisotropy as well as increases in the response magnitudes.

In Fig 12 the result of computing surface representations from initial orientation sensitive filtering and subsequent grouping to create a sketch-like shape representation are shown. The boundary and self-occlusion lines here are enhanced by the feedback from the orientation contrast processed in module III and the rest of the image locations are inhibited. From this representation depth occlusion cues such as T-junctions could be extracted, by applying junction detectors to the 2D sketch representation with a detection process such as in [31]. Then a map of the summed aligned texture energy from module II is shown. The higher the value, the more anisotropic the texture appears. These anisotropies refer to locations of local slant in the surface orientation relative to the observer view point. From the distribution of orientation responses generated by the filters in module II, an estimation of the slant and tilt of the surface can be determined. Fig 13 shows three patches in the luminance function of the texture patterns as examples of the calculation using Eqs (3), (4), (5) and (6) to generate an estimation of anisotropy, tilt orientation and amount of slant from the information in the distribution of summed module II activity over frequency per orientation, shown as histograms.

Most previous approaches stop at this stage, generating needle maps from these estimations, that is, a cartesian representation with a vector at every point defined as perpendicular to the object surface plane. This is enough for simple object shapes such as spheres and cylinders with regular textures. However, for complex shaped objects with highly anisotropic textures, these approaches fail and a global integrating process is needed to give a direct estimation of depth order. In this contribution, such a process is proposed based on the neural operations of filtering and normalization.

**Fig 14:**
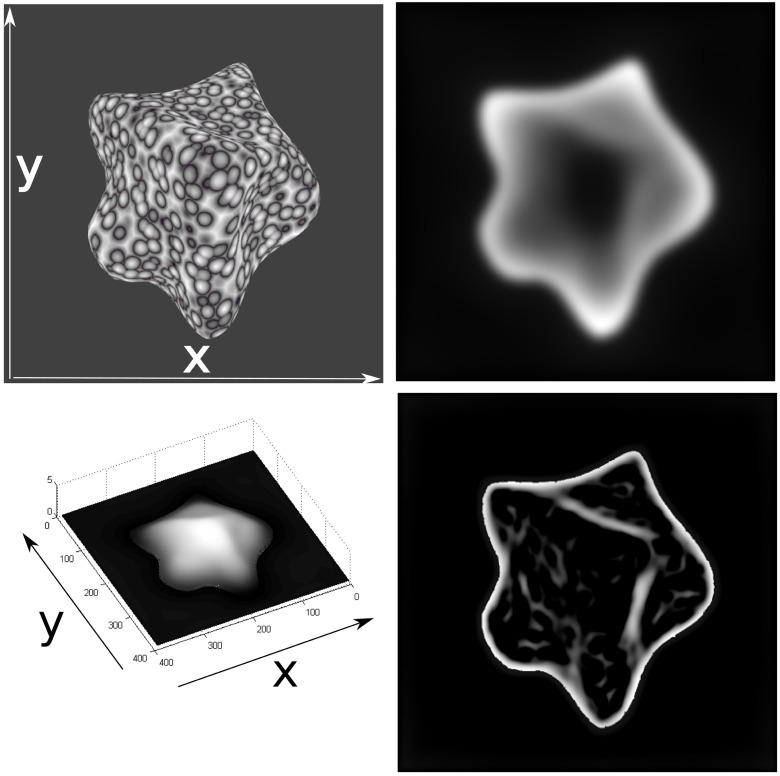
Surface relative depth results for an artificial image. Final result (top right)—Masked module IV response activity matrix (far → white, near → black). Inverted final result mesh (bottom left) seen in perspective. Resulting activity distribution for the sketch representation generated by module III (bottom right).

**Fig 15:**
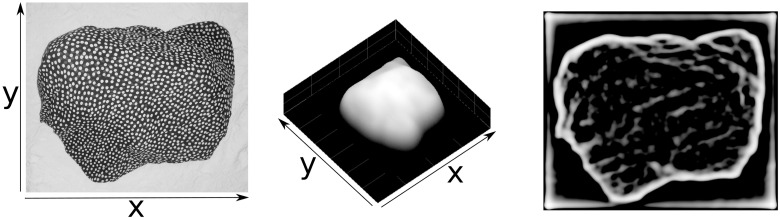
Relative surface depth estimates for a real image. An input intensity image is shown on the left. In the middle panel the inverted summed result of module IV responses is shown. The result of computing the sketch after enhancement is shown on the right.

Results from the surface depth output for an artificial image are shown in Fig 14. The image is a perspective projection of the object and therefore it is meant only to be a qualitative result showing the correct depth ordering of the object, since the model does not compensate for changes and distortions produced by the perspective projection, impeding a direct comparison of profiles such as is done for the image taken under orthographic projections below. Fig 15 shows the result for a real image. In order to exclude regions not belonging to the object from processing, a mask is applied to the image. This mask was drawn manually for the real image, but it is assumed that a neural process of figure-ground segregation could perform this region selection mechanism prior to qualitative depth processing. This process could be performed on the basis of the 2D sketch through a process like the one proposed in [32], where figure-ground segregation is performed in an architecture modeling cortical areas V1 to IT. In that model, given a shape outline, V2 cells compute hypotheses on the direction of border ownership, which are then disambiguated by feedback signals from area IT cells, in a recurrent architecture.

**Fig 16:**
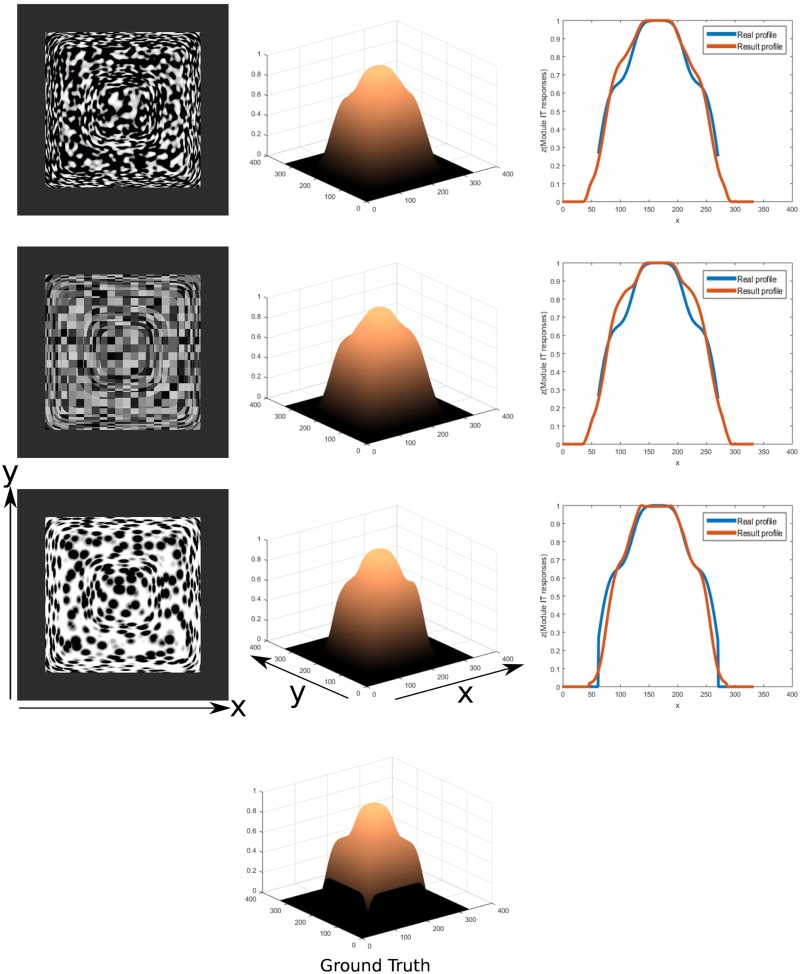
3D depth structure computed for different input textures for the same surface geometry (left). Results of inferred depth structure are shown (center) for given ground truth pattern (bottom). Relative error measures are calculated to determine the deviation of depth estimation from the true shape (7.66%, 9.51% and 6.7% respectively, from top to bottom). Profiles of a section at the middle x coordinate(right) of the inferred depth structure(red line) and compared to the ground truth depth profile(blue line).

In Fig 16 the results of orientation sensitive integration of texture gradient responses that lead to a viewer-centric surface depth representation are shown. These results are compared against the ground truth surface height map in order to demonstrate the invariance of the inferred shape independent of the texture pattern in the input.

The model result is inverted along the z direction by subtracting the z values from the maximum of all these values representing depth and normalized to 1 by dividing by that maximum. The same is calculated for the ground truth height map to be able to compare them. A mask for the object is used to exclude regions that do not belong to the object. The projection is scaled orthographically.

In order to quantify how good the model results fit with the original 3D test mesh, a relative error is computed according to the following equations:

$$\begin{array}{c} E=\frac{\sum_{i}\left|o_{i}-m_{i}\right|}{\sum_{i}o_{i}} \end{array}$$

where *o*_*i*_ is the value of the original test mesh at point *i* and *m*_*i*_ is the value for the model result at that location. This is done in order to compare what are the differences in relation to the depth values of the original object.

**Fig 17:**
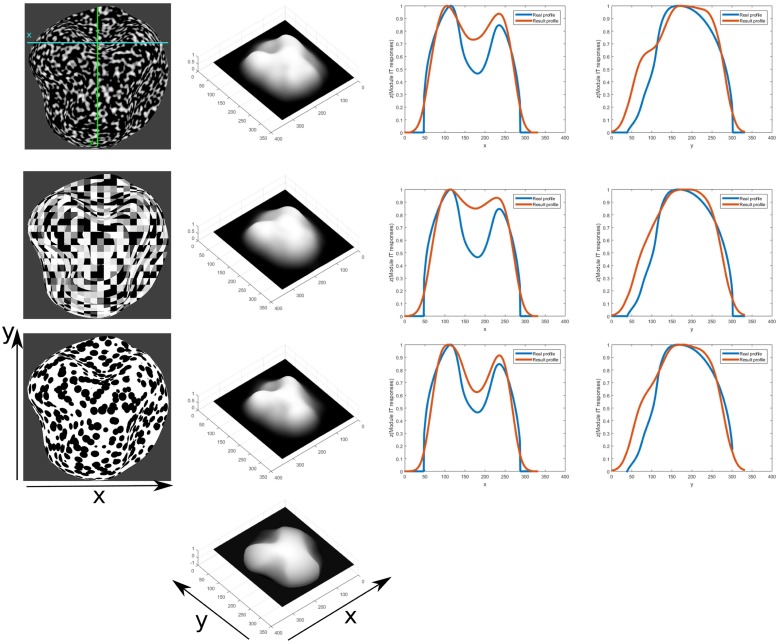
3D depth structure computed for different input textures for the same surface geometry of a doubly curved object(first column). Results of inferred depth structure are shown (second column) for given ground truth pattern (bottom). Relative error measures are calculated to determine the deviation of depth estimation from the true shape(17.23%, 21.99% and 13.76% respectively for x profile; 17.91%, 16.92% and 17.57% respectively for y profile, from top to bottom). Profiles of a cut at the middle x(cyan line) and y(green line) image coordinate(last two columns) of the inferred depth structure(red line) and compared to the ground truth depth profile(blue line).

**Fig 18:**
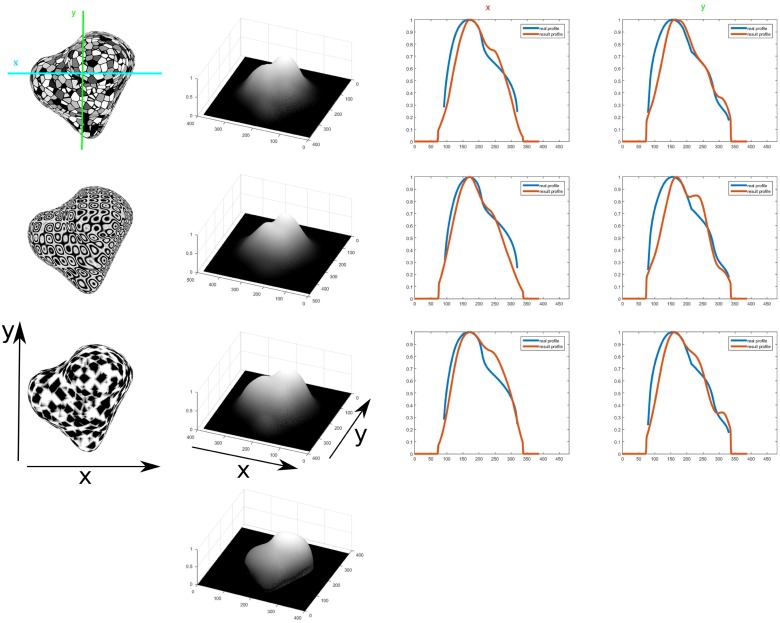
Results and profiles for an object structure featuring multiple consecutive levels of depth for different textures. The profiles come from the horizontal line section x(cyan) and vertical line section y(green) marked in the top left image. Relative error measures are 16.23%, 15.42% and 16.42% respectively, for x profile(red); 16.2%, 17.12% and 14.1%, respectively for y profile, from top to bottom. At the bottom row second column the ground truth 3D mesh of the object is shown.

The profiles for the mid horizontal lines of the test object meshes and the results of the model for the different textures are compared in Fig 16. It can be seen that the results fit well and give a correct shape similarity with a few specks of noise. In some cases the depth is underestimated, but the shape of the profile result is still close to the original, even under the noisy conditions of the textures. It can be seen that for the convex geometry with a convex bump in the middle the steep surface inclinations are underestimated by the model (Fig 16 and surface slants at the object in Fig 17). For the doubly curved surfaces the modulations of the concavities are underestimated such that the model perceptual appearance results in shallower surface variations. These effects cohere qualitatively with human perception [33, 34]. Profiles along horizontal and vertical sections in the texture images are compared to the ground truth image. The relative differences are higher than in the previous results mostly due to the underestimation of the surface depth. The general shape of the profile as well as the location of minima and maxima fit closely to the ground truth profile. Results for an object featuring consecutive levels of depth are shown in Fig 18 for a different set of textures. In the profiles can be seen that the levels of depth are properly detected. Most of the differences result from underestimating the depth level and the resulting profiles having a narrower horizontal extension than the ground truth profile, resulting in a large difference in height when compared.

**Fig 19:**
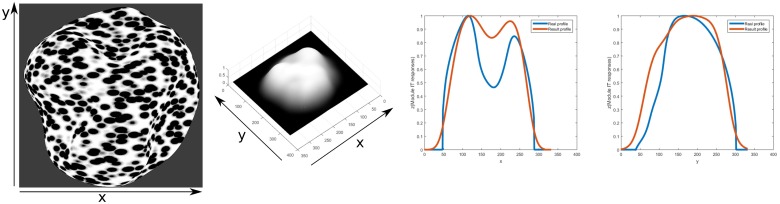
3D depth structure computed for input anisotropic dots texture for the same surface geometry in Fig 17 of a doubly curved object(first column). Results of inferred depth structure are shown (second column). Relative error measures are calculated to determine the deviation of depth estimation from the true shape(22.88%for x profile and 20.60% for y profile). Profiles of a cut at the same x and y coordinate for this object(last two columns) of the inferred depth structure are shown with a red line and compared to the ground truth with a blue line.

**Fig 20:**
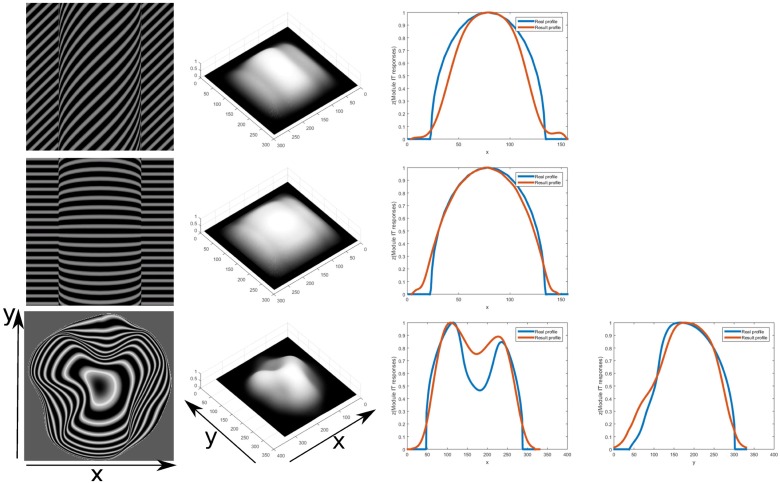
3D depth structure computed for input contour texture for a cylinder and a doubly curved object(first column). Results of inferred depth structure are shown (second column). The first row correspond to a cylinder using contour lines diagonal with respect to the object axis at 45 degrees under perspective projection with 40 degrees field view. The same applies for the second row but using horizontal lines. Profiles for the middle horizontal coordinate for the cylinder are shown in their corresponding third column. The third row corresponds to the same doubly curved object with contour lines texture(Fig 17). Relative error measures for this object are calculated to determine the deviation of depth estimation from the true shape(16.61%for x profile and 15.24% for y profile). Profiles of a cut at the same x(cyan line) and y(green line) coordinates(last two columns) of the inferred depth(red line) are shown compared to the ground truth(blue line).

Fig 19 deals with the case of an anisotropic dot texture. Compression of the texture elements in this case causes them to look less anisotropic in the slanted areas along the larger axis of the texture element, which causes confusion in models which assume an isotropic shape of the texture element. The results for the profile of the object correctly convey the concavities and convexities of the ground truth. The model is successful in such textures since the gradients are on the total orientation energy relative to neighboring energies. The total summed activity from module II would be almost the same when compared to neighboring areas, giving a low or zero activity from the Gaussian derivative filters. The summed activity from module II is still higher on the slanted areas and a depth change can be captured (compare Fig 13, second and third row activity). Results for another difficult texture are shown in Fig 20, with a contour texture. Orientation contrast, relative spectral energy and its integration are proposed here to be factors in the successful perception of this type of textures. Psychophysical studies such as [33, 35] provide evidence that the human visual system is capable of an accurate perception of surface extrema or curvature, even though there is debate on the underlying mechanisms that generate this perception. The first row shows results for diagonal lines for a cylinder. The profile looked narrower in horizontal extension when compared to the ground truth. There was a steeper fall-off at the sides of the cylinder object, due to higher orientation contrast in those areas. The second row shows results for horizontal lines. The shape of the profile has a good fit to the ground truth. It can be seen in the 3D mesh for this texture that the middle part is slightly higher than the rest, due to less orientation contrast in that area. The profiles are normalized to one for the comparison however and it can be notice from the 3D mesh that with horizontal lines the result is shallower when compared to the diagonal lines, due to there being less orientation contrast and relative frequency change. It could be necessary information from the shape of the contours to give a more accurate estimation of the shape. This model does not include this information. The last row shows the same complex object from before but with a contour texture. Even when the profiles’ depths are underestimated, the general shape resembles closely that of the ground truth. That is, qualitatively the concavities and convexities of the shape are captured to generate a surface representation that correctly matches the input surface geometry. The locations of extrema are also represented at their proper locations.

**Fig 21:**
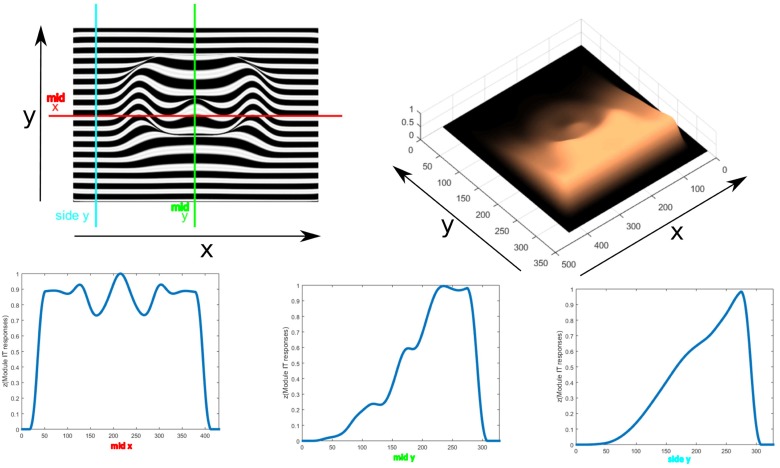
3D depth structure computed for input contour texture for a doubly curved object on an inclined plane with respect to view position. Results of inferred depth structure are shown (top right). The complex object using horizontal contour lines is on a plane slanted 45 degrees under orthographic projection. Profiles of a section at the middle x(red line), middle y(green line) and side y(cyan) coordinates(bottom three columns in that order) of the inferred depth structure are shown.

**Fig 22:**
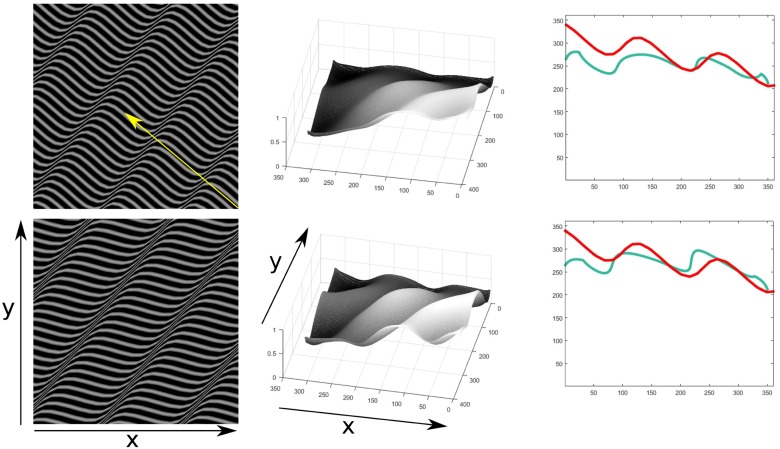
3D depth structure computed for input contour textures in different orientations for an inclined sinusoidal plane oriented 45 degrees around the X axis, 30 around the Y axis and 15 around the Z axis, under orthographic projection. Results of inferred depth structure are shown (second column). Profiles of a cut at the middle vertical coordinates along the depth of the object are shown on the third column. The ground truth profile is plotted in red and the corresponding result in green. The yellow arrow indicates the chosen direction of integration.

Estimating shape from contour or planar cuts textures for objects on inclined planes is difficult for many computational approaches, yet such images are perceptually compelling for shape for humans under the right viewing conditions. In Fig 21 the results for such a texture for the model proposed here are shown. The object and texture is similar to left of Fig 12 depicted in [36]. In this case however, it is necessary for the model to assume a direction of integration. For this image the assumed direction is vertical and only one of the module IV IT fields is given a high weight. Some changes to the parameters for the orientation contrast module and gradient integration modules where also necessary. The receptive field size was increased, in order to better capture the orientation contrast on regions of low frequency and to ensure the integration along the whole object and plane. The results are only claimed to be qualitatively correct, further processing higher in the hierarchy of the visual cortex may deal with the reversing of the projection of the object on the inclined plane. Our model fails to capture the shape of the object in the case of more complex inclined planes such as the one in Fig 22. In this case the plane is sinusoidal and oriented at different angles around the X, Y and Z axes. The integration in this case is performed along the direction oriented at 40 degrees clockwise from the horizontal axis, as an estimation perpendicular to the wave fronts of the sinusoidal depiction in the image. It does not capture however the inclination of the ground truth profile. The model is thus not complete to deal with such textures and in addition to finding ways of choosing how the integration should be performed, it also might need to incorporate geometric information on the contours of the texture, to account for human perception, such as it has been investigated in [37]. The use of orientation contrast information together with spatial frequency information is then proposed here as a complementary model for the perception of such textures.

## Discussion

A neural model is proposed that allows to generate 3D relative depth shape representation of a complex textured object. The model architecture utilizes a hierarchical computational scheme of different modules referring to cortical areas V1, V2, V4 and IT along the ventral pathway to generate representations of 3D shape. The main contributions in this article are:

- The proposal of a mechanistic hierarchical model for generating a neural representation of 3D shape of complex object surfaces. The model generates a direct mesh of depth values. The model achieves this by only using combined canonical neural operations like filtering, normalization and integration in a feed-forward and feed-back processing. Although it is here simplified the model hints at a process of texture gradient integration within cortical area IT.
- A method for the estimation of slant and tilt from orientation response distributions is also proposed. Each orientation response of model V2 cells summed along the frequency domain is considered as a the magnitude of a force oriented at that particular orientation in the complex plane. The magnitude and argument of the resulting sum of forces gives the inclination and orientation of the surface.
- The model also generates a 2D surface sketch from texture images through multi-scale orientation contrast detection. The 2D sketch contains depth cues such as T-junctions or occlusion boundaries as well as ridge-like structures depicting lines of minimum surface curvature.

Unlike previous approaches the model goes beyond a simple detection of local energies of oriented filtering to explain how such localized responses are integrated into a coherent depth representation. Also it does not rely on a heuristic scale-to-depth mapping, like LIGHTSHAFT, to assign relative depth to texture gradients, and also does not require diffusive filling-in of a depth quantity (steered by a boundary web representation). Instead, responses distributed anisotropically in the orientation feature domain are selectively integrated for different orientations and spatial frequencies to generate qualitative depth. In sum, the model hierarchy assumes different filtering operations which compute first and second order changes within different feature domain representations and ranges therein. Thus, the architecture resembles principles to extract invariant properties from the optic array as proposed by [38] (see [39] for a discussion). Unlike approaches to metric quantitative estimation of 3D surface properties we here emphasize the computation of gradient structure utilizing the three-stage cascaded computations in each of the model cortical areas employed [34]. Through feedforward signal propagation combined with modulatory feedback and normalization such filtering operations become nonlinear to selectively highlight the features in the spatial orientation-frequency domain representations linked to qualitative surface shape.

### Relation to previous models

Visual texture can assume different component structure which suffers from compression along the direction of surface slant when the object appearance curves away from the viewer’s sight. Texture gradients provide a potent cue to local relative depth [40]. Several studies have investigated how size, orientation or density of texture elements convey texture gradient information [41]. Evidence suggests that patterns of changing energy in the distribution of filter responses convey the basic information to infer shape from texture that needs to be integrated along characteristic intrinsic surface properties [7].

Previous computational models try to estimate surface orientation from distortions of the apparent optical texture in the image. The approaches can be subdivided according to their task specificity and the computational strategies they use. Geometric approaches are suggested to reconstruct the structure of the metric surface geometry (e.g., [42–44]). In [45] a model was developed which estimates shape and orientation parameters of an affine transformation between neighboring image patches. The model assumes stationarity under translations and that the gaussian curvature of the surface does not change too much. To measure texture distortion a local spectrogram is computed for a patch and compared to the spectrogram of a neighboring patch. Such spectral differences are used to estimate the parameters of the affine transformation.

Neural models, on the other hand, infer the relative or even ordinal structure from initial spatial frequency selective filtering, subsequent grouping of the resulting output responses and a depth mapping step [46, 47]. The LIGHTSHAFT model of [47] utilizes scale-selective initial orientation filtering and subsequent long-range grouping. Relative depth in this model is inferred by depth-to-scale mapping associating coarse-to-fine filter scales to depth using orientation sensitive grouping cells which define scale-sensitive spatial compartments to fill-in qualitative depth.

Grouping mechanisms can be utilized to generate a raw surface sketch to establish lines of minimal surface curvature as a ridge-based qualitative geometry representation [4]. Changes in orientation spectral energy [7, 46] can be integrated to derive local maps of relative surface orientation. Such responses may be integrated to generate globally consistent relative depth maps from such local energy gradient responses.

Similar to the LIGHTSHAFT model [47] the architecture proposed here computes initial spatial frequency-selective responses and subsequently groups them into internal boundaries. Scale-specific boundary web representations in the LIGHTSHAFT model form surface internal barriers for a subsequent filling-in process that generates surface qualities utilizing a scale-to-depth mapping logic. Unlike this approach we employ frequency-related response normalization such that relative frequency energy in different channels provide direct input for gradient estimation. Subsequent grouping and competition between filter responses leads to enhanced representations of intrinsic qualitative surface properties, such as self-occlusions, junctions, minimum curvature ridges to extract a sketch-like representation of the surface structure [4]. The model is not only capable of estimating tilt and slant of the object surface, where many of the previous approaches stop but also to give a holistic representation of a complex multiple depths texture object.

In [48] a model that relates contour curvature to surface curvature on developable surfaces (surfaces with zero Gaussian curvature) is presented. This model requires the resolution of a differential equation for the computation of the local surface normal and is restricted to developable surfaces and contours along geodesics of the surface. In [37] a model is proposed that is based on three free parameters for slant, tilt and scaling factor for contours as planar cuts. Based on the estimates of these parameters, possible estimations of the 3D shapes can be given. This model is not restricted to developable surfaces. For these types of textures our model uses the orientation contrast, frequency compression and subsequent directed gradient integration, instead of explicitly dealing with the contour properties of the texture. The model requires only a direction of integration in this case, aside from that the other computations are done mechanistically. The model, however, needs to be further developed to account for human perception of contour textures, such as the shearing and scaling effects in the experiments in [37].

### Limitations of the model

One of the shortcomings of the current model for the case of the 3D surface representation is the assumption of a defined figure-ground segregation of the object. In principle the 2D sketch result could be used to generate the required segregation, with already established models of the neural process for figure-ground segregation. For example, the model of [32] might provide such framework. Along a hierarchical structure of feed-forward and feed-back processing, oriented contrasts are grouped into contours and shape curvatures are estimated. Finally, border ownership is assigned to curved contour segments such that foreground figures can be labeled and separated from the background. It will be left for future extension to the model to fully integrate all these different functionalities. The current model has a bias toward finding convex regions propagating integrated texture energy gradients changes toward the object borders. The current model however gives plausibility of a gradient integration process along the ventral stream starting in cortical area V1 and culminating in area IT. It can be speculated that this process is not limited to texture gradients but that also other types of features, since there is evidence that area IT could also include other depth cues such as shading [34]. The model is currently only partially able to deal with contour textures, since for inclined surfaces it requires to define a direction or directions of integration as well as include other sources of information such as the geometric properties of the contours for a complete perception.

## Supporting Information

S1 File Receptive fields and individual module equations. (PDF)

S1 Fig Receptive fields (filters) profiles. (TIF)

S1 Table Parameters values used in the equations. (PDF)
