# Supplementary material for: Biologically Inspired Model for Inference of 3D Shape from Texture
Source: PLoS One. 2016 Sep 20;11(9):e0160868. doi: 10.1371/journal.pone.0160868 (PMC5029942; doi:10.1371/journal.pone.0160868)
Supplement: S1 File — (PDF) [file pone.0160868.s001.pdf]

APPENDIX

A Receptive Fields

Each stage from the model architecture performs its filtering stage through a set of filters, resembling the receptive fields of neurons in the visual cortical areas to which it is mapped. Here are presented the equations for such filters as well as any operation performed on their responses.

A.1 Module I equations

The raw image $\mathbb{I}$ is filtered with a complex Gabor filter normalized to sum to 1.

$$G_{\omega,\theta}(I) = \left\| \left(\frac{1}{2 \cdot \pi\sigma^2} \cdot \exp \frac{-(x'^2 + \kappa^2 \cdot y'^2)}{2 \cdot \sigma^2} \cdot \exp(2 \cdot \pi \cdot \frac{1}{\lambda} \cdot x') \right) \star \mathbb{I} \right\|, \qquad \text{(A.1.1a)}$$

$$x' = x \cdot \cos(\theta) + y \cdot \sin(\theta), \qquad \text{(A.1.1b)}$$

$$y' = -x \cdot \sin(\theta) + y \cdot \cos(\theta) \qquad \text{(A.1.1c)}$$

where $\star$ denotes convolution. Each filter is tuned to one of 5 Wavelengths $\lambda = 2 - 32$ pixels logarithmically scaled and to one of 8 Orientations $\theta = 0 - 157.5$ degrees, with the aspect ratio $\kappa = 1$ and a bandwidth of 1 octave (see Figure 1a).

G is normalized according to:

$$F(\mathbb{I}) = G_{\omega,\theta,i} = \frac{G_{\omega,\theta,i}}{(\alpha_G \cdot \hat{G_\omega}) + G_{\omega,i}} \qquad \text{(A.1.2)}$$

where $G_\omega = \sum_\theta G_{\omega,\theta}$, and $\hat{G_\omega} = G_\omega \star \Lambda_\omega$, with Λ_ω a normalized gaussian with $\sigma = 20 \cdot [0.5611 - 7.5493]$
$\alpha_G = 2.5$.

A.2 Module II equations

The receptive fields of cortical area V2 are modeled in module II as two gaussian filter lobes separated a distance τ from each other along their main axis

$$g_{Left}(\omega,\theta) = \frac{1}{2 \cdot \pi\sigma_x(\omega)\sigma_y(\omega)} \exp\left(-\left(\frac{(x' - \tau(\omega))^2}{2 \cdot \sigma_x(\omega)^2} + \frac{y'^2}{2 \cdot \sigma_y(\omega)^2}\right)\right), \qquad \text{(A.2.1a)}$$

$$g_{Right}(\omega,\theta) = \frac{1}{2 \cdot \pi\sigma_x(\omega)\sigma_y(\omega)} \exp\left(-\left(\frac{(x' + \tau(\omega))^2}{2 \cdot \sigma_x(\omega)^2} + \frac{y'^2}{2 \cdot \sigma_y(\omega)^2}\right)\right) \qquad \text{(A.2.1b)}$$

Each lobe is convoluted with its corresponding response in frequency and orientation from area I and multiplicatively combined:

$$F(r^I_{i,\omega,\theta}) = (r^I_{i,\omega,\theta} \star g_{left}(\omega,\theta)) \cdot (r^I_{i,\omega,\theta} \star g_{Right}(\omega,\theta)) \qquad \text{(A.2.2)}$$

The parameters for each lobe are $\sigma_x(\omega) = 22 \cdot d(\omega), \sigma_y = 6 \cdot d(\omega), \tau = 12 \cdot d(\omega)$, where d is an array of logarithmically scaled values in [0.56 - 7.5493] corresponding to each ω frequency channel. The lobes are tuned to 8 orientations $\theta = 0 - 157.5$ degrees (see Figure 1b).

A.3 Module III equations

The input to module III is formed by the sum in frequency channel per orientation of the responses from stage II.

$$\mathbb{I}^{III}_{i,\theta} = \sum_{\omega} r^{II}_{i,\omega,\theta} \tag{A.3.1}$$

The receptive fields in module III correspond to those in cortical area V4, formed by 3 parallel anisotropic gaussian filters laterally offset by a distance τ, for corresponding frequency channel ω and orientation θ:

$$\Lambda_{center}(\omega,\theta) = \mathbb{I}^{III}_{\theta} \star K \cdot \exp(-(\frac{x'^2}{2\cdot\sigma_x^2} + \frac{y'^2}{2\cdot\sigma_y^2})), \tag{A.3.2a}$$

$$\Lambda_{left}(\omega,\theta) = \mathbb{I}^{III}_{\theta} \star K \cdot \exp(-(\frac{x'^2}{2\cdot\sigma_x^2} + \frac{(y'-\tau)^2}{2\cdot\sigma_y^2})), \tag{A.3.2b}$$

$$\Lambda_{right}(\omega,\theta) = \mathbb{I}^{III}_{\theta} \star K \cdot \exp(-(\frac{x'^2}{2\cdot\sigma_x^2} + \frac{(y'+\tau)^2}{2\cdot\sigma_y^2})) \tag{A.3.2c}$$

and combined:

$$Z^{III}{}_{i,\omega,\theta}(\mathbb{I}^{III}_{i,\theta}) = \sum_{\hat{\theta}} [\Lambda_{center}(\omega,\hat{\theta}) - c\cdot\Lambda_{left}(\omega,\hat{\theta})]_+ + [\Lambda_{center}(\omega,\hat{\theta}) - c\cdot\Lambda_{right}(\omega,\hat{\theta})]_+ \tag{A.3.3}$$

Bank of filters for 5 log scales ($\sigma_x = 2\cdot[0.5611 - 1.77], \sigma_y = 0.5\cdot[0.5611 - 1.77], \tau = 1\cdot[0.5611 - 1.77]$ and 8 orientations $[0 - 157.5]$)(see Figure 1c).

A.4 Module IV equations

The input received in area IV is the sum in frequency ω and orientation θ.

$$\mathbb{I}^{IV}_{i} = \sum_{\omega,\theta} r^{II}_{i,\omega,\theta} \tag{A.4.1}$$

The filtering is done in two phases. The first phase with difference of offset gaussian filters for 5 log scales ($\sigma = 4\cdot[0.5611 - 3.77]$ and 8 orientations $[0 - 157.5]$)(see Figure 1d).

$$\Lambda_{left}(\omega,\theta) = \mathbb{I}^{IV}_{\theta} \star K \cdot \exp(-(\frac{x'^2}{2\cdot\sigma_x^2} + \frac{(y'-\tau)^2}{2\cdot\sigma_y^2})), \tag{A.4.2a}$$

$$\Lambda_{right}(\omega,\theta) = \mathbb{I}^{IV}_{\theta} \star K \cdot \exp(-(\frac{x'^2}{2\cdot\sigma_x^2} + \frac{(y'+\tau)^2}{2\cdot\sigma_y^2})), \tag{A.4.2b}$$

$$\Lambda'{}_{\omega,\theta} = \Lambda_{left}(\omega,\theta) - \Lambda_{right}(\omega,\theta), \tag{A.4.2c}$$

$$G'{}_{\omega,\theta} = \mathbb{I}^{IV} \star \Lambda'(\omega,\theta) \tag{A.4.2d}$$

The second phase takes the corresponding output from the first phase and filters it with two long range filter components 2D gaussians weighting functions multiplied by

left/right directed 2D sigmoideal functions (see Figure 1e).

$$\Lambda_{-}(\omega,\theta) = -K \cdot \exp(-(\frac{x'^2}{2\cdot\sigma_x^2} + \frac{y'^2}{2\cdot\sigma_y^2})) \cdot \Psi_{-}(\theta), \tag{A.4.3a}$$

$$\Lambda_{+}(\omega,\theta) = K \cdot \exp(-(\frac{x'^2}{2\cdot\sigma_x^2} + \frac{y'^2}{2\cdot\sigma_y^2})) \cdot \Psi_{+}(\theta), \tag{A.4.3b}$$

$$\Psi_{-}(\theta) = \frac{1}{1+\exp(-A\cdot(x')-B)}, \tag{A.4.3c}$$

$$\Psi_{+}(\theta) = \frac{1}{1+\exp(A\cdot(x')-B)} \tag{A.4.3d}$$

The parameters for these filters are: $\sigma_x = 12 \cdot [7.5493]$, $\sigma_y = 1 \cdot [7.5493], A = 3, B = 7$

$$L(\omega,\theta) = G'_{\omega,\theta} \star \Lambda_{-}(\omega,\theta), \tag{A.4.4a}$$

$$R(\omega,\theta) = G'_{\omega,\theta} \star \Lambda_{+}(\omega,\theta), \tag{A.4.4b}$$

$$Z^{IV}{}_{\omega,\theta} = max(L(\omega,\theta), R(\omega,\theta)) \tag{A.4.4c}$$

Since the gradient calculation will have a sign, the bipolar fields also have a sign, such that they match the sign and direction of integration. Taken the maximum allows to select the path moving away from the peaks of the surface. This would give a tendency to have a convex object bias. The profiles for the bipole fields can be seen in Figure 1f.

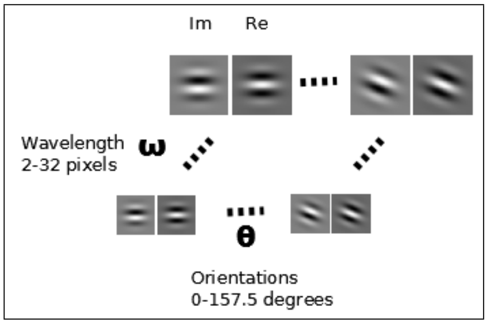

(a) Module I filterbank

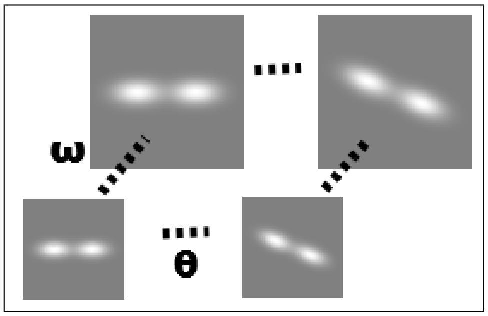

(b) Module II filterbank

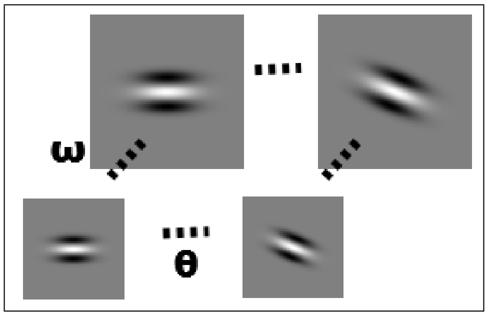

(c) Module III filterbank

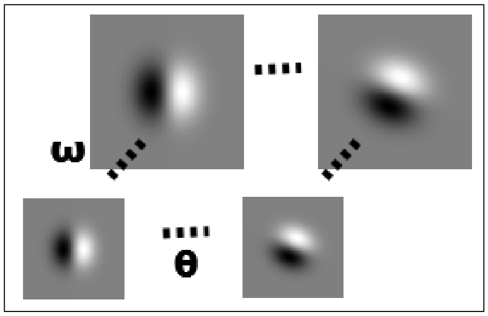

(d) Module IV filterbank - First phase, gradient extraction

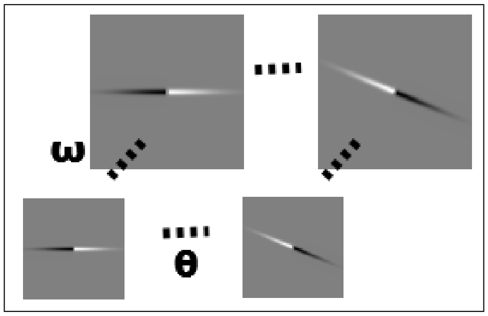

(e) Module IV filterbank - Second phase, gradient integration

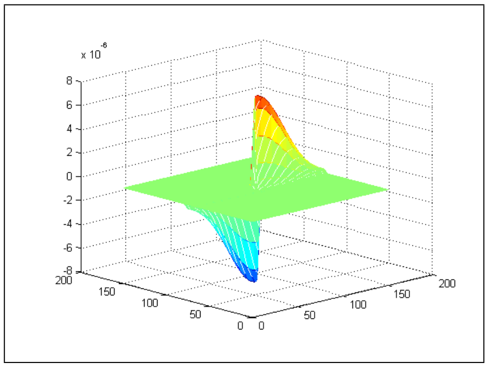

(f) Module IV Bipole Fields Profiles

Figure 1. Filters used in each module. Each one resembles receptive fields for neuron cells in the ventral pathway. Module I uses pairs of complex Gabor filters for local frequency decomposition resembling area V1 receptive fields. Module II uses a pair of Gaussian filters for grouping in area V2. Module III uses three alternating positive-negative Gaussian filters to detect orientation contrast and resemble receptive fields in V4. Likewise module IV uses a pair of positive-negative Gaussian filters to extract the gradient and then an elongated and truncated pair of positive-negative Gaussian filters for gradient integration.

B Model Equations

B.1 Steady State Excitatory Equations

Module I

$$r^{I}_{i,\omega,\theta} = \frac{\beta \cdot f(F(\mathbb{I})) \cdot (1 + net^{I,FB}_{i,\omega,\theta}) - \xi \cdot q^{I,in}_{i,\omega,\theta} + \eta}{\alpha + \gamma \cdot f(F(\mathbb{I})) \cdot (1 + net^{I,FB}_{i,\omega,\theta}) + q^{I,in}_{i,\omega,\theta}} \tag{B.1.1a}$$

Module II

$$r^{II}_{i,\omega,\theta} = \frac{\beta \cdot f(F(r^{I}_{i,\omega,\theta})) \cdot (1 + net^{II,FB}_{i,\omega,\theta}) - \xi \cdot q^{II,in}_{i,\omega,\theta} + \eta}{\alpha + \gamma \cdot f(F(r^{I}_{i,\omega,\theta})) \cdot (1 + net^{II,FB}_{i,\omega,\theta}) + q^{II,in}_{i,\omega,\theta}} \tag{B.1.2a}$$

Module III

$$r^{III}_{i,\omega,\theta} = \frac{\beta \cdot f(Z^{III}{}_{i,\omega,\theta}(\mathbb{I}^{III}_{i,\theta})) \cdot (1 + net^{III,FB}_{i,\omega,\theta}) - \xi \cdot q^{III,in}_{i,\omega,\theta} + \eta}{\alpha + \gamma \cdot f(Z^{III}_{i,\omega,\theta}(\mathbb{I}^{III}_{i,\theta})) \cdot (1 + net^{III,FB}_{i,\omega,\theta}) + q^{III,in}_{i,\omega,\theta}} \tag{B.1.3a}$$

Module IV

$$r^{IV}_{i,\omega,\theta} = \frac{\beta \cdot f(Z^{IV}{}_{i,\omega,\theta}(\mathbb{I}^{IV})) \cdot (1 + net^{IV,FB}_{i,\omega,\theta}) - \xi \cdot q^{IV,in}_{i,\omega,\theta} + \eta}{\alpha + \gamma \cdot f(Z^{IV}_{i,\omega,\theta}(\mathbb{I}^{IV})) \cdot (1 + net^{IV,FB}_{i,\omega,\theta}) + q^{IV,in}_{i,\omega,\theta}} \tag{B.1.4a}$$

B.2 Inhibitory Equations

Module I

$$q^{I,in}_{i,\omega,\phi} = \delta \cdot (\sum_{\phi} r^{I}_{i,\omega,\phi} + \epsilon \cdot \sum_{j} \max_{\omega,\phi}(r^{I}_{j,\omega,\phi}) \cdot \Lambda^{pool}_{ij}) \tag{B.2.1a}$$

Module II

$$q^{II,in}_{i,\omega,\theta} = \delta \cdot (\sum_{\phi} r^{II}_{i,\omega,\phi} + \epsilon \cdot \sum_{j} \max_{\phi}(r^{II}_{j,\omega,\phi}) \cdot \Lambda^{pool}_{ij}) \tag{B.2.2a}$$

Module III

$$q^{III,in}_{i,\omega,\theta} = \delta \cdot (\sum_{\phi} r^{III}_{i,\omega,\phi} + \epsilon \cdot \sum_{j} \max_{\phi}(r^{III}_{j,\omega,\phi}) \cdot \Lambda^{pool}_{ij}) \tag{B.2.3a}$$

Module IV

$$q^{IV,in}_{i,\omega,\theta} = \delta \cdot (\sum_{\phi} r^{IV}_{i,\omega,\phi} + \epsilon \cdot \sum_{j} \max_{\phi}(r^{IV}_{j,\omega,\phi}) \cdot \Lambda^{pool}_{ij}) \tag{B.2.4a}$$

B.3 Feedback Equations

To Module I

$$net^{I,FB}_{i,\omega,\theta} = [\lambda_{FB} - r_{i,\omega,\theta}{}^{II}]_{+} \cdot \sum_{j,\omega,\theta} r^{II}_{j,\omega,\theta} \cdot \Lambda^{pool_{FB}}_{ij} \tag{B.3.1a}$$

To Module II

$$net^{II,FB}_{i,\omega,\theta} = F^{III} + F^{IV} \tag{B.3.2a}$$

with $F^{III} = [\lambda_{FB} - r_{i,\omega,\theta}{}^{III}]_{+} \cdot \sum_{j,\omega,\theta} r^{III}_{j,\omega,\theta} \cdot \Lambda^{pool_{FB}}_{ij}$ and $F^{IV} = |\lambda_{FB} - r_{i,\omega,\theta}{}^{IV}|_{+} \cdot \sum_{j,\omega,\theta} r^{IV}_{j,\omega,\theta} \cdot \Lambda^{pool_{FB}}_{ij}$

B.4 2D Sketch Calculation

The 2D sketch is computed from the total sum in frequency and orientation of the output responses from Module II.

$$R^{II}_{s} = \left[\left(\sum_{\omega,\theta} r^{II}_{i,\omega,\theta} \right) \star \Lambda_{-} \right]_{+} \tag{B.4.1}$$

,where $\Lambda_{-}(\omega)$ is a difference of gaussian with $\sigma_{center} = 2$ and $\sigma_{surround} = 6$

and then normalized

$$R_s^{II} = \frac{R_s^{II}}{(\alpha_R \cdot \hat{G_\omega}) + R_s^{II}} \tag{B.4.2}$$

where $\hat{G_\omega} = R_s^{II} \star \Lambda_\omega$, with Λ_ω a gaussian with $\sigma = 40 \cdot [7.5493]$ and $\alpha_R = 10.5$.

B.5 Surface 3D Mesh Calculation

The surface 3D representation is computed by summing all orientation output responses from each frequency band from Module IV.

$$R_\omega^{IV} = \sum_\theta r_{i,\omega,\theta}^{IV} \tag{B.5.1}$$

Each frequency band is the normalized.

$$R_\omega^{IV} = \frac{R_\omega^{IV}}{(\alpha_R \cdot \hat{G_\omega}) + R_\omega^{IV}} \tag{B.5.2}$$

where $\hat{G_\omega} = R_\omega^{IV} \star \Lambda_\omega$, with Λ_ω a normalized gaussian with $\sigma = 40 \cdot [7.5493]$

The final result is

$$R = \sum_\omega R_\omega^{IV} \tag{B.5.3}$$

C Parameters

	α	β	ξ	η	γ	δ	ϵ	λ_{FB}
Area I	1	1	0.0001	0	1	0.2	0.1	-
Area II	1	1	0.0001	0	1	0.2	0.1	1.1
Area III	1	1	0.00001	0	1	0.2	0.1	1.1
Area IV	1	1	0.00001	0	1	0.2	0.1	1.1

Table 1. This table gives the value for the parameters of the above equations
