# Supplementary figures and images for: Biologically Inspired Model for Inference of 3D Shape from Texture
Source: PLoS One. 2016 Sep 20;11(9):e0160868. doi: 10.1371/journal.pone.0160868 (PMC5029942; doi:10.1371/journal.pone.0160868)

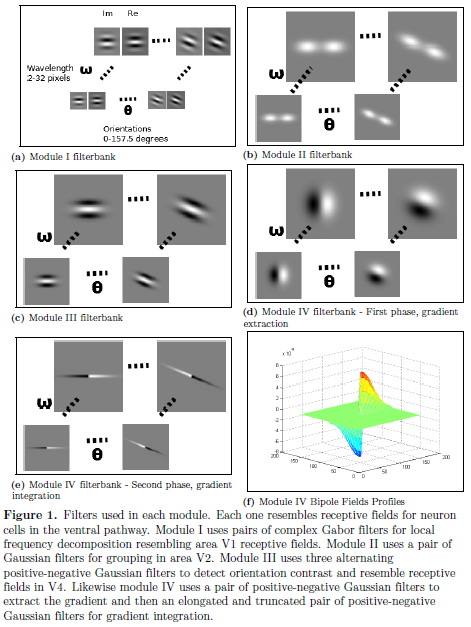

Supplement: S1 Fig — (TIF) [file pone.0160868.s002.tif]
