# Supplementary material for: Biologically Inspired Model for Inference of 3D Shape from Texture
Source: PLoS One. 2016 Sep 20;11(9):e0160868. doi: 10.1371/journal.pone.0160868 (PMC5029942; doi:10.1371/journal.pone.0160868)
Supplement: S1 Table — (PDF) [file pone.0160868.s003.pdf]

1 Parameters

	α	β	ξ	η	γ	δ	ϵ	λ_{FB}
Area I	1	1	0.0001	0	1	0.2	0.1	-
Area II	1	1	0.0001	0	1	0.2	0.1	1.1
Area III	1	1	0.00001	0	1	0.2	0.1	1.1
Area IV	1	1	0.00001	0	1	0.2	0.1	1.1

Table 1. This table gives the value for the parameters of the above equations
